# Crystal structure and Hirshfeld surface analysis of [(*R*,*S*)-2,8-bis­(tri­fluoro­meth­yl)quinolin-4-yl](piperidin-2-yl)methanol methanol monosolvate

**DOI:** 10.1107/S2056989025006310

**Published:** 2025-07-23

**Authors:** Dennis Awasabisah, Navamoney Arulsamy, Mason Primrose, Guoxing Lin

**Affiliations:** a Biology and Chemistry Department Fitchburg State University, 160 Pearl St Fitchburg MA 01420 USA; b Department of Chemistry University of Wyoming,Laramie WY 82071 USA; c Gustaf H. Carlson School of Chemistry, Arthur M. Sackler Sciences Center, 950 Main Street, Worcester, MA 01610, USA; Universität Greifswald, Germany

**Keywords:** crystal structure, mefloquine, DFT, anti­malarial, quinoline, Hirshfeld surface analysis, inter­molecular hydrogen bonds

## Abstract

The title compound, C_17_H_16_F_6_N_2_O·CH_3_OH, is composed of a quinolinyl group and a piperidinyl group connected *via* a hy­droxy­methine (–CHOH) functionality. The compound, which is monosolvated by methanol, was crystallized *via* slow evaporation of a methanol solution, yielding colorless prism-like crystals. The hy­droxy­methine center of the compound is in the absolute *R* configuration, whereas the chiral center of the piperidinyl ring is in the *S* configuration. The conformation of the piperidinyl ring is a chair. The supra­mol­ecular architecture of the crystal is sustained by a set of hydrogen bonds: O—H⋯O, N—H⋯O and C—H⋯F. The inter­molecular forces are further analyzed and confirmed by a Hirshfeld surface analysis. DFT structural data computed with the *ORCA* quantum chemistry program package using the B3LYP/def2-TZVPP basis set compare quite well with the experimental X-ray crystal structural data.

## Abstract 2

The compound [(*R*,*S*)-2,8-bis­(tri­fluoro­meth­yl)quinolin-4-yl](piperidin-2-yl)methanol methanol monosolvate crystallizes in the *I*4_1_/*acd* space group. The X-ray crystal structure determination revealed that O—H⋯O, N—H⋯O and O—H⋯N hydrogen bonds are involved in the inter­molecular forces of attraction. This is supported by Hirshfeld surface analysis. The X-ray crystallographic data are supported by DFT calculations.

## 1. Chemical context

Quinoline derivatives such as quinine, mefloquine and chloro­quine are biologically relevant compounds that have a range of applications (Matada *et al.*, 2021 ▸). For example, when they serve as anti­malarials, these compounds target heme during the hemozoin formation process in the blood stage level of the life cycle of the *Plasmodium* parasite (Gorka *et al.*, 2013 ▸ and references therein). These quinoline-based drugs are believed to inter­act directly with heme to generate a heme–drug adduct, thus inhibiting hemozoin (Gorka *et al.*, 2013 ▸). We have shown in previous work that quinine coordinates to the ruthenium center of a heme model compound, (OEP)Ru(CO) (OEP = 2,3,7,8,12,13,17,18-octa­ethyl­porphyrinato; Awasabisah *et al.*, 2024 ▸). In that report, we obtained the crystal structure of a quinoline–ruthenium porphyrin complex, (OEP)Ru(CO)(Qnl), which confirmed the coordination of the quinoline nitro­gen atom to the ruthenium center. In a follow-up to that investigation, we aimed to study the reactions of other quinoline-based compounds with synthetic heme model complexes. During these studies we obtained a crystal structure of mefloquine, a synthetic analogue of quinine. The compound crystallized as the absolute (−)mefloquine isomer (*i.e. R*,*S*-mefloquine).

## 2. Structural commentary

**Figure 1:**
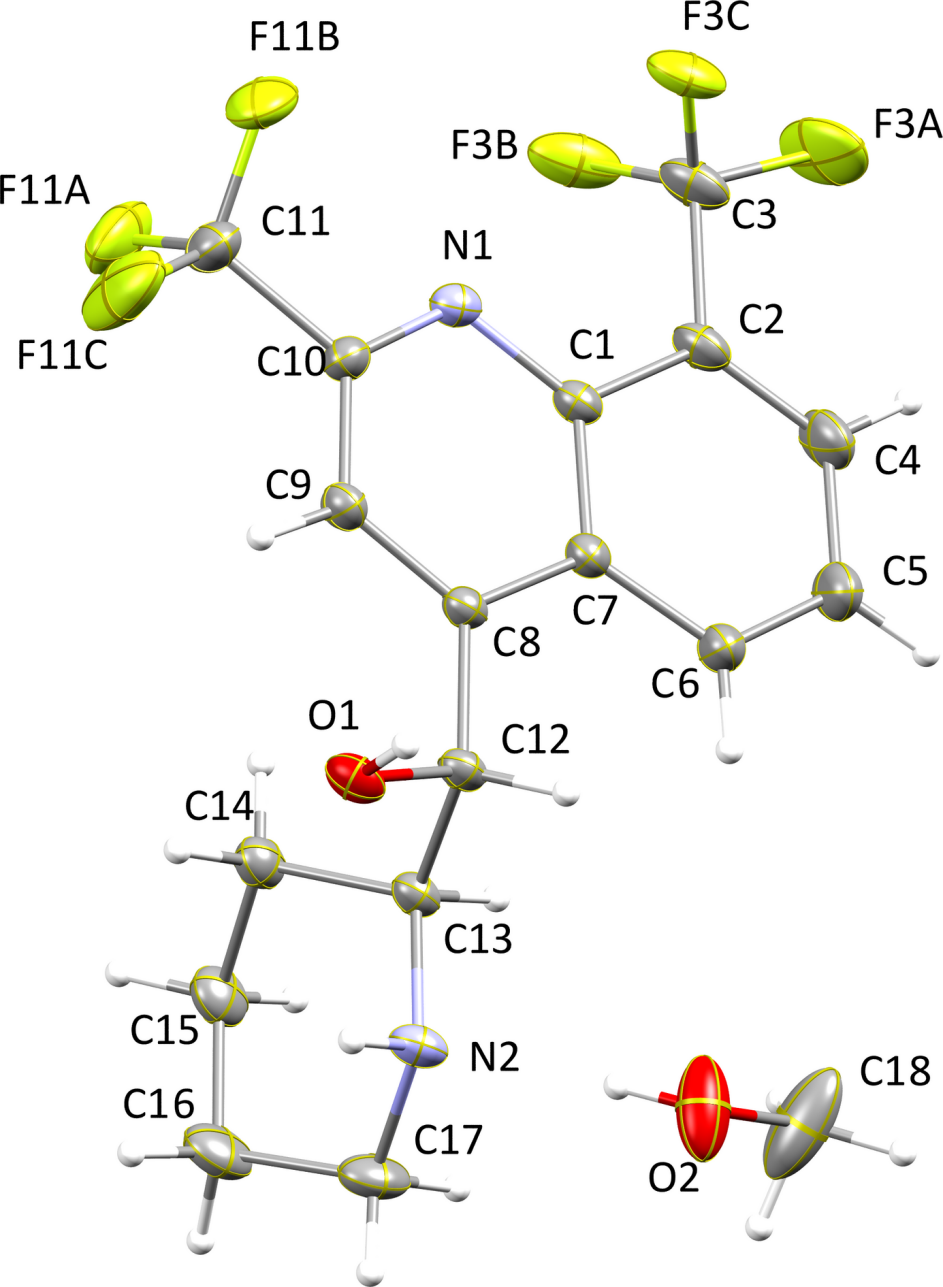
Mol­ecular structure of the title compound. Displacement ellipsoids are drawn at the 50% probability level.

The title compound crystallizes in the tetra­gonal *I*4_1_/*acd* space group with one mefloquine and one methanol mol­ecule in the asymmetric unit and *Z* being 32. Previously reported structures for *rac*-mefloquine with no methanol solvate (Skórska, *et al.*, 2006 ▸) and the structures of chiral (−)mefloquine and (+)mefloquine crystallized in contrast in centrosymmetric monoclinic (*P*2_1_/*n*) and non-centrosymmetric ortho­rhom­bic (*P*2_1_2_1_2_1_) space groups (Dassonville-Klimpt *et al.*, 2013 ▸). The mol­ecular structure of the title compound is shown in Fig. 1 ▸.
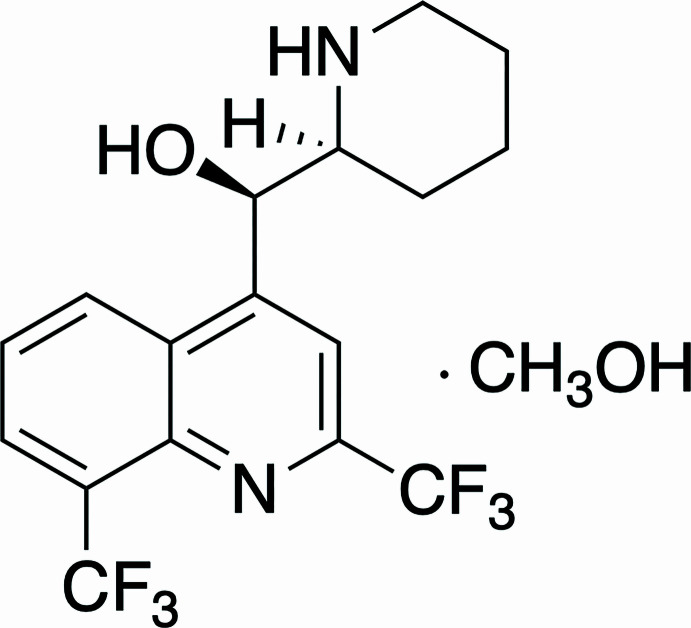


The quinolinyl ring and the piperidinyl ring in mefloquine are linked *via* a hy­droxy­methine moiety. The hy­droxy­methine carbon center and the nitro­gen center of the piperidinyl group are in the absolute *R* and absolute *S* configurations, respectively. As expected, the quinolinyl group is planar, whereas the piperidinyl ring exhibits a chair conformation. The nitro­gen atom of the latter, N2, is pyramidalized with the sum of the three angles being 328.4°. The torsional angles involving the two rings, *i.e.* C8—C12—C13—N2 and C8—C12—C13—C14, are 179.7 (1) and −55.7 (1)°, respectively. The geometry at the hy­droxy­methine center, as expected, is tetra­hedral with the C8—C12—C13 bond angle of 111.62 (10)° being the largest, and the O1—C12—C13 bond angle, 107.89 (10)°, being the smallest.

**Figure 2:**
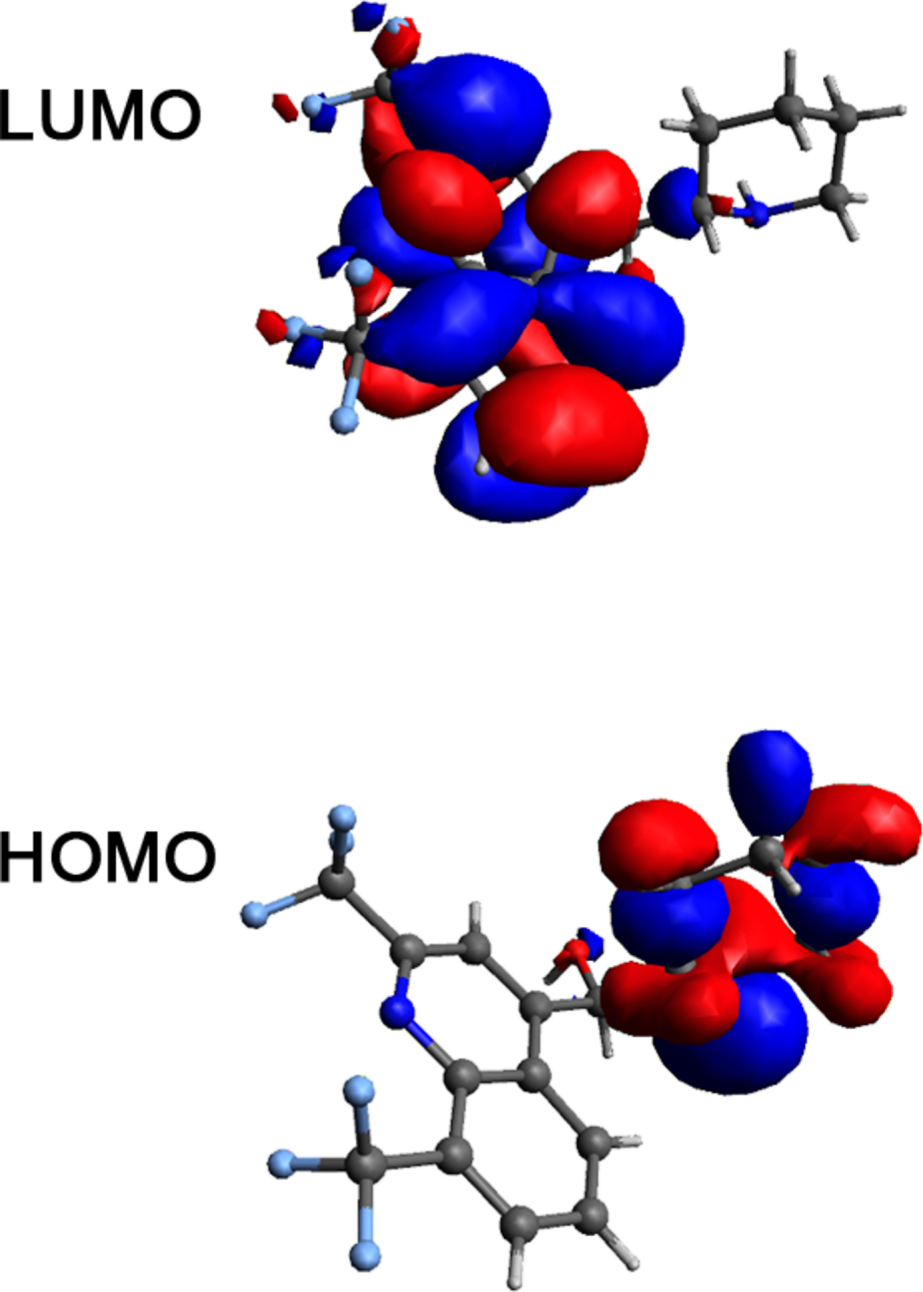
The computed frontier mol­ecular orbitals of the title compound.

The title compound was also subjected to DFT computations. The structure was geometrically optimized with the *ORCA* program using the B3LYP/def2-TZVPP basis set (Neese, 2012 ▸, 2022 ▸; Neese *et al.*, 2020 ▸). The coordinates obtained by X-ray diffraction were used as an input file. The methanol solvate, however, was not included in the calculation. The experimentally determined bond lengths obtained by X-ray crystallography are well in agreement with those obtained from the optimized structure (see supporting information). For example, the quinolinyl C—N bond lengths obtained by X-ray crystallography are 1.3575 (17) and 1.3070 (17) Å, and their respective values determined by DFT calculations are 1.352 and 1.306 Å, which constitutes an excellent agreement. The piperidinyl C—N bond lengths determined by X-ray crystallography are 1.4719 (16) and 1.4740 (17) Å, and their DFT values are slightly shorter at 1.465 and 1.464 Å, respectively. The quinolinyl and piperidinyl C—N—C bond angles are 116.43 (11) and 112.43 (11)°, respectively. Their corresponding DFT values are 118.23 and 113.08°. DFT calculations (B3LYP/def2-TZVPP) of the frontier mol­ecular orbitals revealed the HOMO to be located largely on the piperidinyl ring, while the LUMO resides on the quinolinyl moiety (Fig. 2 ▸).

## 3. Supra­molecular features

**Figure 3:**
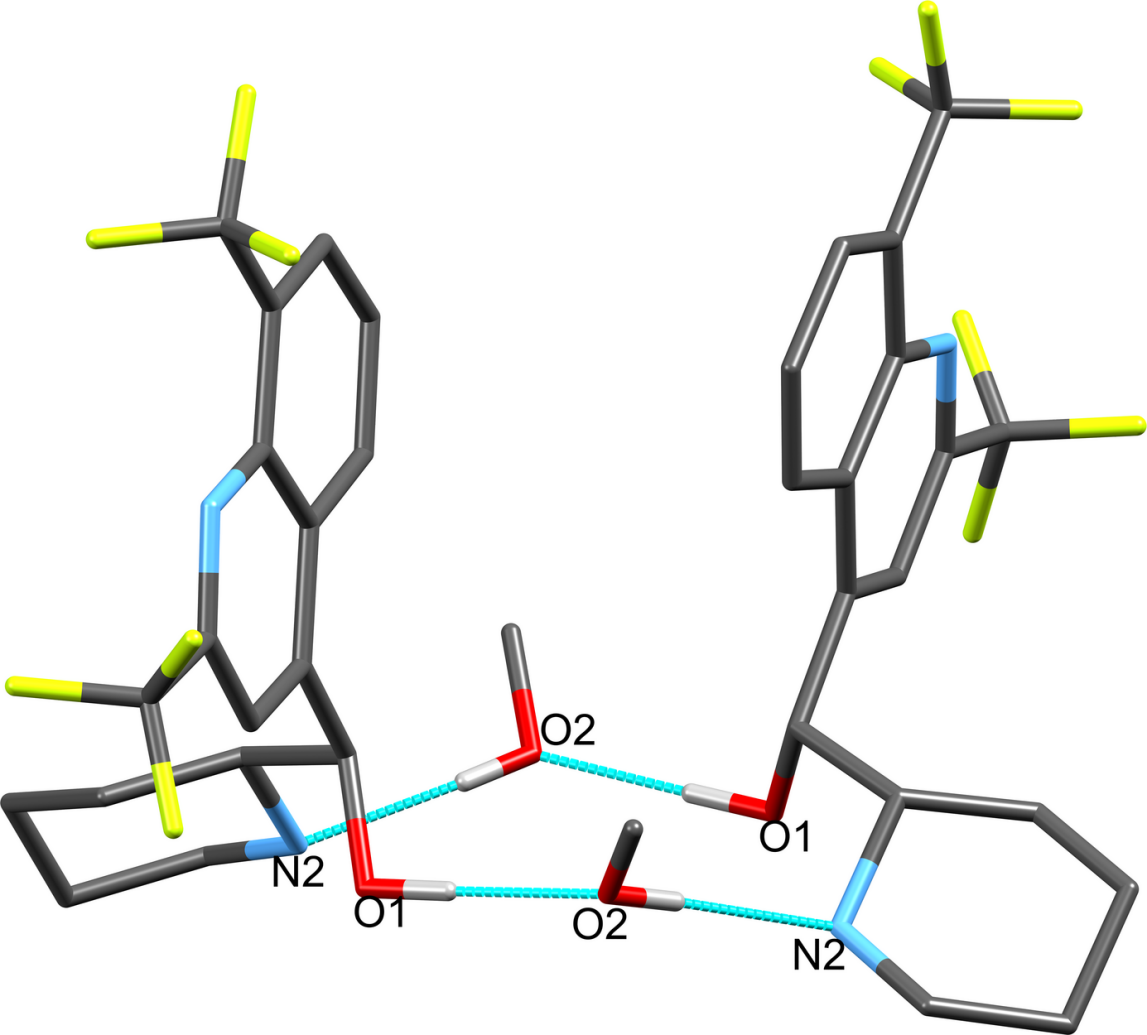
Hydrogen-bonding inter­actions between the hy­droxy atoms of the hy­droxy­methine moiety and the methanol solvate and the piperidinyl nitro­gen atom. H atoms not involved in the hydrogen bonds are omitted for clarity. Symmetry codes: −*x* + 1, −*y* + 1, −*z* + 1 (methanol at the front); *x*, *y* − 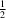, −*z* + 1 (mefloquine to the right).

Apart from crystallizing in a space group of particularly high symmetry in contrast to previously reported structures, further notable differences can be observed in the inter­molecular hydrogen-bonding inter­actions (Table 1 ▸). The present structure exhibits hydrogen-bonding inter­actions between the hy­droxy­methine O1/H atoms and atom O2 of the methanol solvate mol­ecule, which then acts as hydrogen-bonding donor to the piperidinyl N2 atom, resulting in a hydrogen-bonding ring structure involving two mefloquine and two methanol mol­ecules (Fig. 3 ▸). In contrast, the structure for *rac*-mefloquine contains arrangements of four mefloquine mol­ecules held directly together by hydrogen bonds involving the hy­droxy­methine O atom and the piperidinyl N atom. The C—O, O—H and N—H bond lengths of the current structure are 1.4144 (15) and 0.898 (2) and 0.85 (2) Å, respectively (Table 1 ▸). In the crystal, the mol­ecules associate *via* O—H⋯O, N—H⋯O, O—H⋯N and C—H⋯F inter­actions.

**Figure 4:**
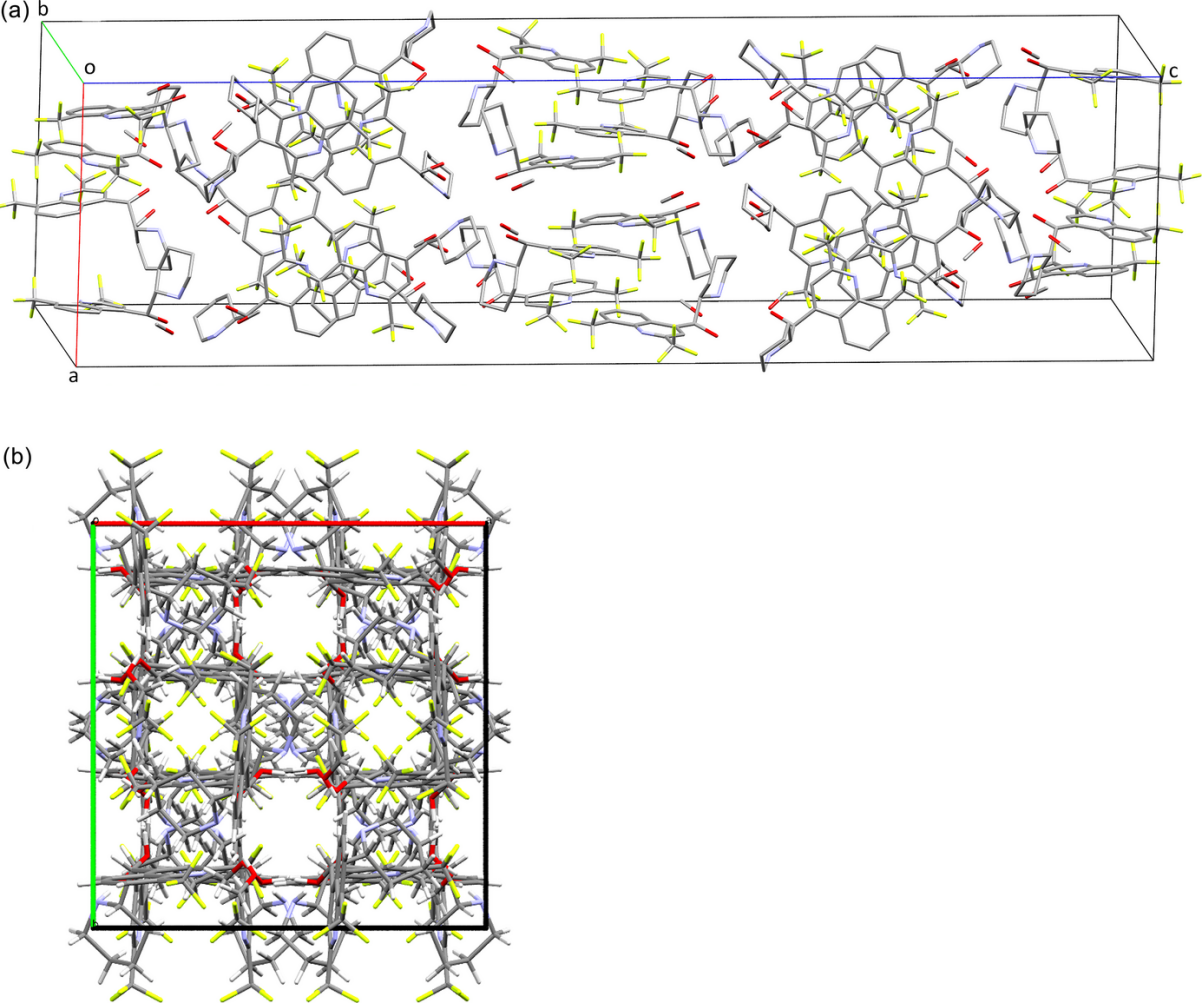
Packing diagram in the crystals of the title compound viewed along the *b* axis (top). The atoms are unlabeled, and all H atoms are omitted for clarity. Below the packing in the crystal shown along the crystallographic *c* axis exhibiting a grid motif (bottom).

Crystal packing diagrams are presented in Fig. 4 ▸. The structures of the chiral mefloquine mol­ecules appear to associate exclusively through inter­molecular hydrogen bonds. The packing pattern consists of stacked ribbons, which alternately protrude through the crystal along the crystallographic *a-* and *b*-axis directions, resulting in grid or chessboard-like structures when viewed along the crystallographic *c* axis. Where the resulting rows at the 0, 1/2, and 1 *x* regions and those at the 1/4 and 3/4 *y* regions cross each other, the structure bears small nearly spherical voids of 114.36 Å^3^ size comprising merely 0.7% of the unit-cell volume.

**Figure 5:**
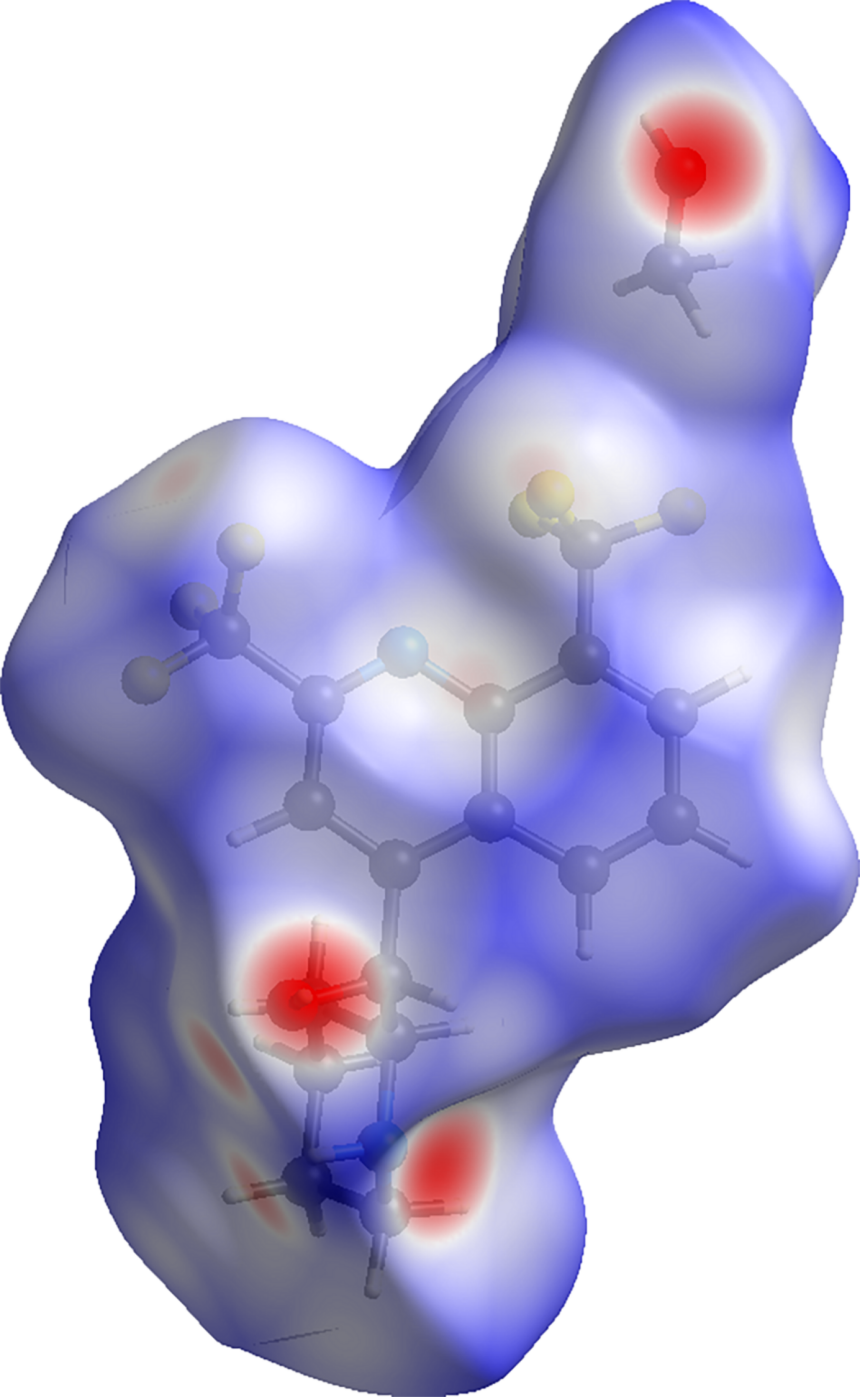
The *d*_norm_ Hirshfeld surface map for the title compound.

**Figure 6:**
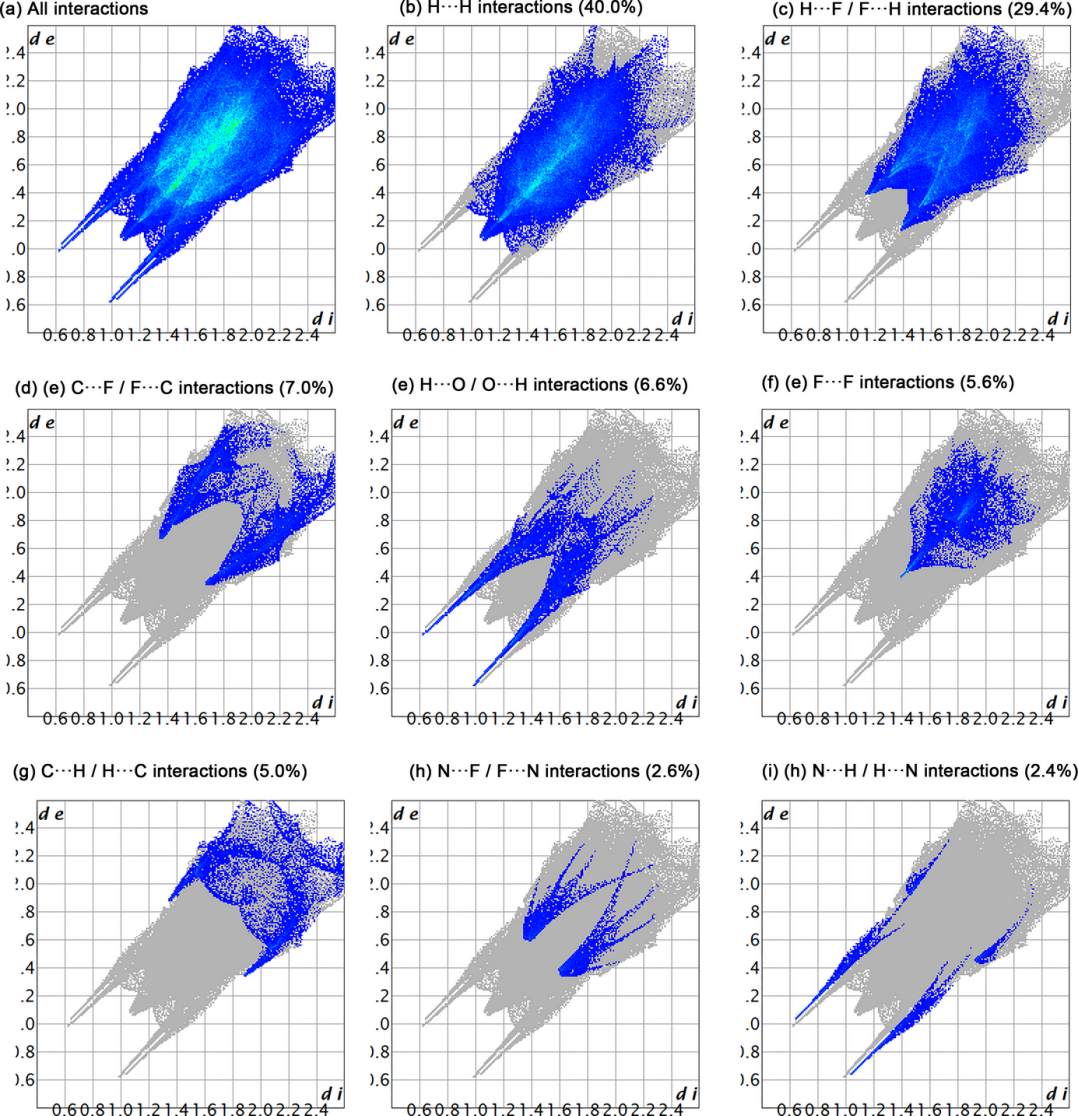
Two dimensional fingerprint plots for the title compound, showing (*a*) all inter­actions, and delineated into (*b*) H⋯H, (*c*) H⋯F/ F⋯H, (*d*) C⋯F/ F⋯C (*e*) H⋯O/ O⋯H, (*g*) C⋯H/ H⋯C, (*h*) N⋯F/ F⋯N, and (*i*) N⋯H/ H⋯N inter­actions with their relative contributions.

In order to gain more insight into the inter­molecular inter­actions among neighboring mol­ecules in the crystal packing of the title compound, a Hirshfeld surface analysis was performed using *CrystalExplorer 21* (Spackman, *et al.*, 2021 ▸). The 3D surface map of the title compound is shown in Fig. 5 ▸. The *d*_norm_ Hirshfeld surface map reveals strong inter­molecular forces (dark-red-colored regions), which are attributed to N⋯H and H⋯O inter­actions involving the piperidinyl N—H, the methanol O—H, as well as the solvated methanol O—H. The *d*_norm_ Hirshfeld surface map also reveals C—H⋯F inter­actions (light red) in the vicinity of the CF_3_ group. The 2D fingerprint plots were assessed to provide qu­anti­tative information about the non-covalent inter­actions in the crystal packing. As revealed by the 2D fingerprint plots (Fig. 6 ▸), the H⋯H and H⋯F/ F⋯H inter­actions are the most prominent, accounting for 40.0% and 29.4%, respectively, of the overall inter­molecular inter­actions. Other notable contributions include, C⋯F/ F⋯C (7.0%), H⋯O/ O⋯H (6.6%), F⋯F (5.6%) and C⋯H/ H⋯C (5.0%). The weakest inter­actions are N⋯F/ F⋯N (2.6%) and N⋯H/ H⋯N (2.4%).

## 4. Database survey

A survey of the Cambridge Structural Database version 2025.1.0 (Groom *et al.*, 2016 ▸; accessed March 2025) using CONQUEST (Bruno *et al.*, 2002 ▸) for the unmodified mefloquine structural motif in cationic or neutral form was carried out. The search returned 16 hit structures of which a large proportion were refined with multiple mol­ecules in their asymmetric units or contain co-crystallized mol­ecules other than solvent and/or counter-ions. Four or even more mol­ecules in the asymmetric unit are found for neutral (*e.g*. QIYREX; Dassonville-Klimpt *et al.*, 2013 ▸) as well as for cationic (*e.g.* BIGTIV; Karle & Karle, 2002 ▸) mefloquine species. A better-defined single-crystal structure of the neutral form with only two mol­ecules in the asymmetric unit is available from the database with refcode LEBYAT (Skórska *et al.*, 2006 ▸). A well representative hydro­chloride form with only a single mol­ecule in the a.u. was reported by Mendes do Prado *et al.* (2014 ▸) (refcode HAJSAO01). All metrical parameters of the title compound and its closely related structures are well comparable and there are not even notable differences between protonated and neutral forms discernible.

## 5. Synthesis and crystallization

Sodium methoxide (137 mg, 2.54 mmol) was placed in a 25 mL Schlenk tube followed by 3 mL MeOH. In a separate vial, mefloquine hydro­chloride (502.0 mg, 1.21 mmol) was dissolved in MeOH (5 mL) then added dropwise to the sodium methoxide solution. The solution was stirred for 3 h during which time it became slightly turbid. The solvent was reduced to *ca.* 5 mL *in vacuo*. The resulting precipitate was filtered under vacuum, and washed with small amounts of cold MeOH. The filtrate was collected and placed in a 10 mL Erlenmeyer flask. A slow evaporation of the filtrate resulted in colorless prism-like crystals suitable for X-ray crystallography. IR (ATR, cm^−1^ intensity): 3411 *br* (*m*), 2949 (*w*), 2920 (*w*), 2856 (*w*), 1641 (*m*), 1602 (*m*), 1431 (*m*), 1381 (*w*) 1367 (*w*) 1305 (*s*), 1265 (*w*), 1210 (*w*), 1185 (*w*), 1104 (*vs*), 1128 (*vs*), 1039 (*m*), 1006 (*w*), 939 (*w*), 890 (*w*), 865 (*w*), 835 (*m*), 768 (*s*), 736 (*w*), 715 (*w*), 686 (*w*), 669 (*m*), 648 (*m*), 616 (*w*). ^1^H NMR (DMSO-*d*_6_, 400 MHz): δ (ppm) δ 8.69 (*d*, *J* = 8.8 Hz, 1H, qnl-*H*), 8.32 (*d*, *J* = 7.2 Hz, 1H, qnl-*H*), 8.06 (*s*, 1H, qnl-*H*), 7.89 (*dd*, *J* = 8.7, 7.2 Hz, 1H, qnl-*H*), 5.93 (*br s*, 1H, O-*H*), 5.29 (*d*, *J* = 5.4 Hz, 1H, C(OH)*H*), 2.67–2.92 (*m*, 2H, pip-*H*), 2.34 (*t*, *J* = 11.1 Hz, 1H, pip-*H*), 1.82 (*br s*, 1H, N—*H*), 1.02–1.32 (*m*, 6H, pip-*H*). ^19^F NMR (DMSO-*d*_6_, 376 MHz,) δ −58.88, −66.64.

## 6. Refinement

Crystal data, data collection and structure refinement details are summarized in Table 2 ▸.

## Supplementary Material

Crystal structure: contains datablock(s) global, I. DOI: 10.1107/S2056989025006310/yz2068sup1.cif

Structure factors: contains datablock(s) I. DOI: 10.1107/S2056989025006310/yz2068Isup2.hkl

Supporting information file. DOI: 10.1107/S2056989025006310/yz2068Isup3.cml

CCDC reference: 2473105

Additional supporting information:  crystallographic information; 3D view; checkCIF report

## Figures and Tables

**Table 1:** Hydrogen-bond geometry (Å, °)

*D*—H⋯*A*	*D*—H	H⋯*A*	*D*⋯*A*	*D*—H⋯*A*
O1—H1⋯O2^i^	0.89 (2)	1.72 (2)	2.6057 (16)	179 (2)
N2—H2*N*⋯O1^ii^	0.85 (2)	2.33 (2)	3.1077 (15)	154.0 (19)
C18—H18*C*⋯F3*A*	0.92 (3)	2.53 (3)	3.193 (2)	129 (2)
O2—H2*O*⋯N2^iii^	0.81 (3)	1.87 (3)	2.6711 (18)	174 (3)

Symmetry codes: (i) 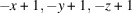; (ii) 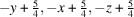; (iii) 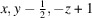.

**Table 2:** Experimental details

Crystal data	
Chemical formula	C_17_H_16_F_6_N_2_O·CH_4_O
*M*_r_	410.36
Crystal system, space group	Tetragonal, *I*4_1_/*acd*
Temperature (K)	100
*a*, *c* (Å)	15.9788 (6), 59.997 (3)
*V* (Å^3^)	15318.6 (14)
*Z*	32
Radiation type	Mo *K*α
μ (mm^−1^)	0.13
Crystal size (mm)	0.44 × 0.40 × 0.36

Data collection	
Diffractometer	Bruker D8 Venture Duo
Absorption correction	Multi-scan (Blessing, 1995 ▸)
*T*_min_, *T*_max_	0.85, 0.95
No. of measured, independent and observed [*I* > 2σ(*I*)] reflections	158826, 5423, 4871
*R*_int_	0.052
(sin θ/λ)_max_ (Å^−1^)	0.696

Refinement	
*R*[*F*^2^ > 2σ(*F*^2^)], *wR*(*F*^2^), *S*	0.053, 0.146, 1.06
No. of reflections	5423
No. of parameters	333
H-atom treatment	All H-atom parameters refined
Δρ_max_, Δρ_min_ (e Å^−3^)	0.65, −0.55

Computer programs: *SAINT* (Bruker, 2019 ▸), *SHELXT2018/2* (Sheldrick, 2015*a* ▸), *SHELXL2019/1* (Sheldrick, 2015*b* ▸), *ShelXle* (Hübschle *et al.*, 2011 ▸) and *publCIF* (Westrip, 2010 ▸).

## supplementary crystallographic information

### [(R,S)-2,8-Bis(trifluoromethyl)quinolin-4-yl](piperidin-2-yl)methanol methanol monosolvate Crystal data

**Table:**

C_17_H_16_F_6_N_2_O·CH_4_O	*D*_x_ = 1.423 Mg m^−^^3^
*M**_r_* = 410.36	Mo *K*α radiation, λ = 0.71073 Å
Tetragonal, *I*4_1_/*a**c**d*	Cell parameters from 9494 reflections
*a* = 15.9788 (6) Å	θ = 2.6–29.4°
*c* = 59.997 (3) Å	µ = 0.13 mm^−^^1^
*V* = 15318.6 (14) Å^3^	*T* = 100 K
*Z* = 32	Prism, colourless
*F*(000) = 6784	0.44 × 0.40 × 0.36 mm

### [(R,S)-2,8-Bis(trifluoromethyl)quinolin-4-yl](piperidin-2-yl)methanol methanol monosolvate Data collection

**Table:**

Bruker D8 Venture Duo diffractometer	4871 reflections with *I* > 2σ(*I*)
Detector resolution: 7.3910 pixels mm^-1^	*R*_int_ = 0.052
ω and φ scans	θ_max_ = 29.7°, θ_min_ = 1.9°
Absorption correction: multi-scan (Blessing, 1995)	*h* = −22→22
*T*_min_ = 0.85, *T*_max_ = 0.95	*k* = −21→22
158826 measured reflections	*l* = −83→83
5423 independent reflections	

### [(R,S)-2,8-Bis(trifluoromethyl)quinolin-4-yl](piperidin-2-yl)methanol methanol monosolvate Refinement

**Table:**

Refinement on *F*^2^	Primary atom site location: dual
Least-squares matrix: full	Hydrogen site location: difference Fourier map
*R*[*F*^2^ > 2σ(*F*^2^)] = 0.053	All H-atom parameters refined
*wR*(*F*^2^) = 0.146	*w* = 1/[σ^2^(*F*_o_^2^) + (0.0776*P*)^2^ + 19.8214*P*] where *P* = (*F*_o_^2^ + 2*F*_c_^2^)/3
*S* = 1.06	(Δ/σ)_max_ = 0.001
5423 reflections	Δρ_max_ = 0.65 e Å^−^^3^
333 parameters	Δρ_min_ = −0.55 e Å^−^^3^
0 restraints	

### [(R,S)-2,8-Bis(trifluoromethyl)quinolin-4-yl](piperidin-2-yl)methanol methanol monosolvate Special details

**Table:**

Geometry. All esds (except the esd in the dihedral angle between two l.s. planes) are estimated using the full covariance matrix. The cell esds are taken into account individually in the estimation of esds in distances, angles and torsion angles; correlations between esds in cell parameters are only used when they are defined by crystal symmetry. An approximate (isotropic) treatment of cell esds is used for estimating esds involving l.s. planes.

### [(R,S)-2,8-Bis(trifluoromethyl)quinolin-4-yl](piperidin-2-yl)methanol methanol monosolvate Fractional atomic coordinates and isotropic or equivalent isotropic displacement parameters (Å^2^)

**Table:**

	*x*	*y*	*z*	*U*_iso_*/*U*_eq_	
N1	0.61574 (7)	0.47489 (7)	0.52401 (2)	0.0173 (2)	
C1	0.62308 (7)	0.55818 (8)	0.52001 (2)	0.0161 (2)	
C2	0.63270 (8)	0.58539 (9)	0.49753 (2)	0.0218 (3)	
C3	0.63376 (9)	0.52166 (10)	0.47904 (2)	0.0270 (3)	
F3A	0.64063 (10)	0.55938 (8)	0.45905 (2)	0.0548 (4)	
F3B	0.69710 (6)	0.46825 (7)	0.48036 (2)	0.0380 (3)	
F3C	0.56412 (6)	0.47602 (6)	0.47807 (2)	0.0308 (2)	
C4	0.63991 (11)	0.66807 (10)	0.49261 (2)	0.0297 (3)	
H4	0.6452 (14)	0.6859 (14)	0.4777 (4)	0.035 (5)*	
C5	0.63740 (11)	0.72894 (10)	0.50962 (2)	0.0304 (3)	
H5	0.6424 (13)	0.7854 (14)	0.5055 (4)	0.035 (5)*	
C6	0.62771 (9)	0.70536 (8)	0.53134 (2)	0.0230 (3)	
H6	0.6259 (13)	0.7465 (13)	0.5425 (3)	0.029 (5)*	
C7	0.62047 (8)	0.61947 (8)	0.53718 (2)	0.0161 (2)	
C8	0.60924 (7)	0.59137 (8)	0.55946 (2)	0.0155 (2)	
C9	0.60064 (8)	0.50698 (8)	0.56305 (2)	0.0184 (2)	
H9	0.5933 (13)	0.4863 (13)	0.5777 (3)	0.030 (5)*	
C10	0.60476 (8)	0.45281 (8)	0.54478 (2)	0.0180 (2)	
C11	0.59709 (11)	0.35957 (9)	0.54840 (3)	0.0277 (3)	
F11A	0.67126 (8)	0.32145 (6)	0.54678 (2)	0.0484 (3)	
F11B	0.54795 (7)	0.32345 (6)	0.53352 (2)	0.0407 (3)	
F11C	0.56779 (11)	0.34104 (7)	0.56857 (2)	0.0599 (4)	
C12	0.60708 (8)	0.65098 (8)	0.57910 (2)	0.0164 (2)	
H122	0.5744 (11)	0.6990 (12)	0.5748 (3)	0.020 (4)*	
O1	0.57190 (6)	0.61217 (7)	0.59810 (2)	0.0214 (2)	
H1	0.5169 (14)	0.6183 (13)	0.5967 (3)	0.030 (5)*	
C13	0.69512 (8)	0.68156 (8)	0.58533 (2)	0.0167 (2)	
H13	0.7168 (10)	0.7145 (11)	0.5729 (3)	0.015 (4)*	
C14	0.75681 (9)	0.61117 (9)	0.59016 (2)	0.0215 (3)	
H14A	0.7634 (12)	0.5749 (12)	0.5774 (3)	0.027 (5)*	
H14B	0.7337 (13)	0.5741 (13)	0.6018 (3)	0.030 (5)*	
C15	0.84104 (9)	0.64723 (10)	0.59736 (3)	0.0269 (3)	
H15A	0.8781 (14)	0.6024 (14)	0.6002 (4)	0.037 (5)*	
H15B	0.8649 (13)	0.6771 (13)	0.5852 (3)	0.030 (5)*	
C16	0.82988 (10)	0.70462 (11)	0.61745 (3)	0.0325 (3)	
H16A	0.8095 (15)	0.6707 (14)	0.6309 (4)	0.039 (6)*	
H16B	0.8838 (16)	0.7324 (17)	0.6214 (4)	0.053 (7)*	
C17	0.76620 (9)	0.77248 (10)	0.61218 (3)	0.0288 (3)	
H17A	0.7554 (14)	0.8067 (13)	0.6255 (4)	0.038 (5)*	
H17B	0.7877 (13)	0.8080 (13)	0.6003 (4)	0.032 (5)*	
N2	0.68546 (7)	0.73889 (7)	0.60436 (2)	0.0197 (2)	
H2N	0.6627 (13)	0.7113 (13)	0.6147 (3)	0.030 (5)*	
C18	0.62912 (17)	0.41400 (13)	0.42266 (5)	0.0577 (7)	
H18A	0.673 (2)	0.396 (2)	0.4292 (7)	0.090 (11)*	
H18B	0.671 (3)	0.436 (3)	0.4125 (9)	0.140 (19)*	
H18C	0.6047 (18)	0.4639 (18)	0.4268 (5)	0.056 (7)*	
O2	0.58920 (8)	0.36933 (10)	0.40650 (3)	0.0453 (4)	
H2O	0.6152 (18)	0.3277 (19)	0.4034 (5)	0.056 (8)*	

### [(R,S)-2,8-Bis(trifluoromethyl)quinolin-4-yl](piperidin-2-yl)methanol methanol monosolvate Atomic displacement parameters (Å^2^)

**Table:**

	*U*^11^	*U*^22^	*U*^33^	*U*^12^	*U*^13^	*U*^23^
N1	0.0157 (5)	0.0189 (5)	0.0173 (5)	−0.0007 (4)	−0.0011 (4)	−0.0035 (4)
C1	0.0150 (5)	0.0203 (5)	0.0130 (5)	−0.0013 (4)	−0.0009 (4)	−0.0012 (4)
C2	0.0235 (6)	0.0293 (7)	0.0125 (5)	−0.0036 (5)	−0.0008 (4)	−0.0017 (5)
C3	0.0266 (7)	0.0402 (8)	0.0142 (6)	−0.0061 (6)	0.0008 (5)	−0.0055 (5)
F3A	0.0955 (10)	0.0575 (7)	0.0115 (4)	−0.0188 (7)	0.0057 (5)	−0.0032 (4)
F3B	0.0197 (4)	0.0582 (7)	0.0362 (5)	0.0018 (4)	0.0018 (4)	−0.0251 (5)
F3C	0.0207 (4)	0.0454 (5)	0.0263 (4)	−0.0039 (4)	−0.0051 (3)	−0.0139 (4)
C4	0.0410 (8)	0.0329 (8)	0.0153 (6)	−0.0069 (6)	−0.0030 (5)	0.0064 (5)
C5	0.0469 (9)	0.0230 (7)	0.0214 (6)	−0.0048 (6)	−0.0062 (6)	0.0065 (5)
C6	0.0320 (7)	0.0181 (6)	0.0191 (6)	−0.0008 (5)	−0.0053 (5)	0.0005 (5)
C7	0.0168 (5)	0.0172 (5)	0.0142 (5)	−0.0002 (4)	−0.0026 (4)	−0.0002 (4)
C8	0.0153 (5)	0.0180 (5)	0.0132 (5)	0.0010 (4)	−0.0016 (4)	−0.0023 (4)
C9	0.0219 (6)	0.0194 (6)	0.0140 (5)	−0.0007 (4)	−0.0003 (4)	0.0005 (4)
C10	0.0198 (5)	0.0144 (5)	0.0197 (6)	0.0001 (4)	−0.0013 (4)	−0.0017 (4)
C11	0.0387 (8)	0.0167 (6)	0.0278 (7)	−0.0016 (5)	−0.0017 (6)	−0.0014 (5)
F11A	0.0455 (6)	0.0213 (5)	0.0784 (9)	0.0100 (4)	−0.0158 (6)	−0.0025 (5)
F11B	0.0463 (6)	0.0221 (4)	0.0536 (6)	−0.0117 (4)	−0.0141 (5)	−0.0033 (4)
F11C	0.1174 (12)	0.0235 (5)	0.0388 (6)	−0.0110 (6)	0.0224 (7)	0.0056 (4)
C12	0.0170 (5)	0.0191 (5)	0.0131 (5)	0.0010 (4)	−0.0010 (4)	−0.0035 (4)
O1	0.0201 (5)	0.0303 (5)	0.0138 (4)	−0.0031 (4)	0.0017 (3)	−0.0024 (3)
C13	0.0156 (5)	0.0207 (5)	0.0137 (5)	0.0008 (4)	−0.0011 (4)	−0.0026 (4)
C14	0.0200 (6)	0.0239 (6)	0.0207 (6)	0.0039 (5)	−0.0029 (5)	−0.0017 (5)
C15	0.0188 (6)	0.0332 (7)	0.0289 (7)	0.0044 (5)	−0.0061 (5)	−0.0005 (6)
C16	0.0237 (7)	0.0428 (9)	0.0310 (7)	0.0007 (6)	−0.0115 (6)	−0.0073 (7)
C17	0.0216 (6)	0.0337 (8)	0.0310 (7)	−0.0034 (5)	−0.0066 (5)	−0.0121 (6)
N2	0.0186 (5)	0.0245 (5)	0.0161 (5)	−0.0021 (4)	−0.0012 (4)	−0.0060 (4)
C18	0.0601 (14)	0.0308 (9)	0.0823 (17)	0.0101 (9)	−0.0426 (14)	−0.0214 (10)
O2	0.0212 (5)	0.0529 (8)	0.0619 (9)	0.0046 (5)	−0.0038 (5)	−0.0334 (7)

### [(R,S)-2,8-Bis(trifluoromethyl)quinolin-4-yl](piperidin-2-yl)methanol methanol monosolvate Geometric parameters (Å, º)

**Table:**

N1—C10	1.3070 (17)	C12—C13	1.5356 (17)
N1—C1	1.3575 (17)	C12—H122	0.962 (19)
C1—C7	1.4220 (16)	O1—H1	0.89 (2)
C1—C2	1.4252 (17)	C13—N2	1.4719 (16)
C2—C4	1.359 (2)	C13—C14	1.5233 (18)
C2—C3	1.5058 (19)	C13—H13	0.974 (17)
C3—F3B	1.3263 (19)	C14—C15	1.526 (2)
C3—F3C	1.3318 (17)	C14—H14A	0.97 (2)
C3—F3A	1.3468 (17)	C14—H14B	0.99 (2)
C4—C5	1.410 (2)	C15—C16	1.525 (2)
C4—H4	0.94 (2)	C15—H15A	0.95 (2)
C5—C6	1.3653 (19)	C15—H15B	0.95 (2)
C5—H5	0.94 (2)	C16—C17	1.520 (2)
C6—C7	1.4213 (18)	C16—H16A	1.03 (2)
C6—H6	0.94 (2)	C16—H16B	1.00 (3)
C7—C8	1.4213 (16)	C17—N2	1.4740 (17)
C8—C9	1.3725 (18)	C17—H17A	0.98 (2)
C8—C12	1.5155 (16)	C17—H17B	0.97 (2)
C9—C10	1.3983 (17)	N2—H2N	0.85 (2)
C9—H9	0.95 (2)	C18—O2	1.363 (2)
C10—C11	1.5106 (19)	C18—H18A	0.85 (4)
C11—F11B	1.3216 (18)	C18—H18B	0.97 (5)
C11—F11C	1.3306 (19)	C18—H18C	0.92 (3)
C11—F11A	1.336 (2)	O2—H2O	0.81 (3)
C12—O1	1.4144 (15)		
			
C10—N1—C1	116.43 (11)	C8—C12—H122	107.8 (11)
N1—C1—C7	122.98 (11)	C13—C12—H122	108.0 (11)
N1—C1—C2	118.41 (11)	C12—O1—H1	105.6 (13)
C7—C1—C2	118.60 (12)	N2—C13—C14	112.33 (10)
C4—C2—C1	120.74 (12)	N2—C13—C12	106.91 (10)
C4—C2—C3	119.78 (12)	C14—C13—C12	113.85 (11)
C1—C2—C3	119.47 (12)	N2—C13—H13	107.0 (10)
F3B—C3—F3C	106.73 (13)	C14—C13—H13	108.3 (10)
F3B—C3—F3A	106.18 (13)	C12—C13—H13	108.1 (10)
F3C—C3—F3A	105.90 (12)	C13—C14—C15	110.22 (12)
F3B—C3—C2	113.58 (12)	C13—C14—H14A	111.3 (12)
F3C—C3—C2	113.16 (12)	C15—C14—H14A	110.8 (12)
F3A—C3—C2	110.75 (13)	C13—C14—H14B	109.6 (12)
C2—C4—C5	120.75 (13)	C15—C14—H14B	110.9 (12)
C2—C4—H4	120.5 (14)	H14A—C14—H14B	103.9 (17)
C5—C4—H4	118.8 (14)	C16—C15—C14	110.34 (12)
C6—C5—C4	120.22 (14)	C16—C15—H15A	112.7 (14)
C6—C5—H5	121.7 (13)	C14—C15—H15A	108.6 (14)
C4—C5—H5	118.0 (13)	C16—C15—H15B	110.6 (13)
C5—C6—C7	120.74 (13)	C14—C15—H15B	108.9 (12)
C5—C6—H6	119.5 (12)	H15A—C15—H15B	105.6 (18)
C7—C6—H6	119.8 (12)	C17—C16—C15	110.04 (12)
C6—C7—C8	123.18 (11)	C17—C16—H16A	109.1 (14)
C6—C7—C1	118.94 (11)	C15—C16—H16A	110.0 (13)
C8—C7—C1	117.87 (11)	C17—C16—H16B	108.1 (16)
C9—C8—C7	118.09 (11)	C15—C16—H16B	110.7 (15)
C9—C8—C12	119.54 (11)	H16A—C16—H16B	109 (2)
C7—C8—C12	122.38 (11)	N2—C17—C16	113.10 (13)
C8—C9—C10	118.70 (11)	N2—C17—H17A	107.9 (14)
C8—C9—H9	120.2 (13)	C16—C17—H17A	110.2 (13)
C10—C9—H9	121.1 (13)	N2—C17—H17B	106.7 (12)
N1—C10—C9	125.92 (12)	C16—C17—H17B	109.4 (13)
N1—C10—C11	114.47 (11)	H17A—C17—H17B	109.5 (18)
C9—C10—C11	119.61 (12)	C13—N2—C17	112.43 (11)
F11B—C11—F11C	107.94 (14)	C13—N2—H2N	107.0 (14)
F11B—C11—F11A	106.18 (13)	C17—N2—H2N	109.4 (14)
F11C—C11—F11A	106.07 (15)	O2—C18—H18A	122 (2)
F11B—C11—C10	112.45 (13)	O2—C18—H18B	94 (3)
F11C—C11—C10	112.25 (12)	H18A—C18—H18B	81 (4)
F11A—C11—C10	111.54 (13)	O2—C18—H18C	116.6 (17)
O1—C12—C8	111.12 (10)	H18A—C18—H18C	121 (3)
O1—C12—C13	107.89 (10)	H18B—C18—H18C	98 (4)
C8—C12—C13	111.62 (10)	C18—O2—H2O	111 (2)
O1—C12—H122	110.4 (11)		

### [(R,S)-2,8-Bis(trifluoromethyl)quinolin-4-yl](piperidin-2-yl)methanol methanol monosolvate Hydrogen-bond geometry (Å, º)

**Table:**

*D*—H···*A*	*D*—H	H···*A*	*D*···*A*	*D*—H···*A*
O1—H1···O2^i^	0.89 (2)	1.72 (2)	2.6057 (16)	179 (2)
N2—H2*N*···O1^ii^	0.85 (2)	2.33 (2)	3.1077 (15)	154.0 (19)
C18—H18*C*···F3*A*	0.92 (3)	2.53 (3)	3.193 (2)	129 (2)
O2—H2*O*···N2^iii^	0.81 (3)	1.87 (3)	2.6711 (18)	174 (3)

Symmetry codes: (i) −*x*+1, −*y*+1, −*z*+1; (ii) −*y*+5/4, −*x*+5/4, −*z*+5/4; (iii) *x*, *y*−1/2, −*z*+1.

**Table:**

Parameter	DFT
N1-C10	1.306
C1-C7	1.430
C2-C4	1.371
C3-F3B	1.344
C3-F3A	1.354
C4-H4	1.080
C5-H5	1.081
C6-H6	1.080
C8-C9	1.374
C9-C10	1.406
C10-C11	1.523
C11-F11C	1.351
C12-O1	1.428
C12-H122	1.093
C13-N2	1.465
C13-H13	1.096
C14-H14A	1.093
C15-C16	1.531
C15-H15B	1.092
C16-H16A	1.095
C17-N2	1.464
C17-H17B	1.096
N1-C1	1.352
C1-C2	1.427
C2-C3	1.514
C3-F3C	1.344
C4-C5	1.409
C5-C6	1.369
C6-C7	1.418
C7-C8	1.426
C8-C12	1.520
C9-H9	1.078
C11-F11B	1.338
C11-F11A	1.347
C12-C13	1.542
O1-H1	0.961
C13-C14	1.537
C14-C15	1.534
C14-H14B	1.092
C15-H15A	1.096
C16-C17	1.532
C16-H16B	1.093
C17-H17A	1.091
N2-H2N	1.014

## References

[bb1] Awasabisah, D., Gangemi, J. F., Powell, D. R. & Lin, G. (2024). *Transit. Met. Chem.* **49**, 75–86.

[bb2] Blessing, R. H. (1995). *Acta Cryst.* A**51**, 33–38. 10.1107/s0108767394005726 7702794

[bb3] Bruker (2019). *SAINT*. Bruker AXS Inc., Madison, Wisconsin, USA.

[bb4] Bruno, I. J., Cole, J. C., Edgington, P. R., Kessler, M., Macrae, C. F., McCabe, P., Pearson, J. & Taylor, R. (2002). *Acta Cryst.* B**58**, 389–397. 10.1107/s0108768102003324 12037360

[bb5] Dassonville–Klimpt, A., Cézard, C., Mullié, C., Agnamey, P., Jonet, A., Da Nascimento, S., Marchivie, M., Guillon, J. & Sonnet, P. (2013). *ChemPlusChem* **78**, 642–646. 10.1002/cplu.201300074 31986621

[bb6] Gorka, A. P., de Dios, A. & Roepe, P. D. (2013). *J. Med. Chem.* **56**, 5231–5246. 10.1021/jm400282d 23586757

[bb7] Groom, C. R., Bruno, I. J., Lightfoot, M. P. & Ward, S. C. (2016). *Acta Cryst.* B**72**, 171–179. 10.1107/S2052520616003954 PMC4822653 27048719

[bb8] Hübschle, C. B., Sheldrick, G. M. & Dittrich, B. (2011). *J. Appl. Cryst.* **44**, 1281–1284. 10.1107/S0021889811043202 PMC3246833 22477785

[bb9] Karle, J. M. & Karle, I. L. (2002). *Antimicrob. Agents Chemother.* **46**, 1529–1534. 10.1128/AAC.46.5.1529-1534.2002 PMC127198 11959592

[bb10] Matada, B. S., Pattanashettar, R. & Yernale, N. G. (2021). *Bioorg. Med. Chem.* **32**, 115973. 10.1016/j.bmc.2021.116098 33740641

[bb11] Mendes do Prado, V., Cardoso Seiceira, R., Pitaluga Jr, A., Andrade-Filho, T., Andrade Alves, W., Reily Rocha, A. & Furlan Ferreira, F. (2014). *J. Appl. Cryst.* **47**, 1380–1386.

[bb12] Neese, F. (2012). *WIREs Comput. Mol. Sci.* **2**, 73–78.

[bb13] Neese, F. (2022). *WIREs Comput. Mol. Sci.* **12**, e1606.

[bb14] Neese, F., Wennmohs, F., Becker, U. & Riplinger, C. (2020). *J. Chem. Phys.* **152**, 224108. 10.1063/5.0004608 32534543

[bb15] Sheldrick, G. M. (2015*a*). *Acta Cryst.* C**71**, 3–8.

[bb16] Sheldrick, G. M. (2015*b*). *Acta Cryst.* C**71**, 3–8.

[bb17] Skórska, A., Śliwiński, J. & Oleksyn, B. J. (2006). *Bioorg. Med. Chem. Lett.* **16**, 850–853. 10.1016/j.bmcl.2005.11.016 16303303

[bb18] Spackman, P. R., Turner, M. J., McKinnon, J. J., Wolff, S. K., Grimwood, D. J., Jayatilaka, D. & Spackman, M. A. (2021). *J. Appl. Cryst.* **54**, 1006–1011. 10.1107/S1600576721002910 PMC8202033 34188619

[bb19] Westrip, S. P. (2010). *J. Appl. Cryst.* **43**, 920–925.

